# Case report: Favorable efficacy of combined afatinib and anlotinib treatment in a lung adenocarcinoma patient harboring uncommon *EGFR* L858M/L861R mutations

**DOI:** 10.3389/fphar.2024.1437086

**Published:** 2024-11-29

**Authors:** Yueming He, Yitao Wu, Rongqi He, Meng Xu, Heshan Chen, Yiran Meng, Liuqing Zheng, Li Wang

**Affiliations:** ^1^ Department of Respiratory and Critical Care Medicine, Quanzhou First Hospital Affiliated to Fujian Medical University, Quanzhou, China; ^2^ Department of Thoracic Surgery, Quanzhou First Hospital Affiliated to Fujian Medical University, Quanzhou, China; ^3^ Department of R&D, Hangzhou Repugene Technology Co., Ltd., Hangzhou, China

**Keywords:** non-small cell lung cancer, rare mutations, EGFR L858M/L861R, afatinib, anlotinib

## Abstract

Targeted therapy has significantly prolonged survival of non-small cell lung cancer (NSCLC) patients carrying common *EGFR* mutations, but the standard care for patients with rare mutations has not been well established. Here, we report a 65-year-old female diagnosed with stage IIIC lung adenocarcinoma located in the right inferior lobe, harboring uncommon *EGFR* L858M/L861R mutations. Remarkably, 24 days post-treatment of afatinib and anlotinib, chest CT scans demonstrated significant shrinkage of primary lesion, indicating a partial response. Except for mild hand-foot syndrome and diarrhea, no other severe adverse symptoms were observed throughout treatment. The patient, now on combination therapy for exceeding 12 months, exhibits further decreased tumor size and a high quality of life. This case underscores the importance of precise molecular diagnosis in guiding therapeutic strategies and provides a valuable reference for clinical decision-making in EGFR-positive NSCLC cases with atypical mutations.

## 1 Introduction

Over the past decades, targeted therapy has revolutionized the clinical treatment of non-small cell lung cancer (NSCLC) worldwide, especially for patients with unresectable tumor lesions. Somatic mutations of epidermal growth factor receptor (*EGFR*) gene are detected in almost 50% of East Asian NSCLC cases and have attracted significant attention in the realm of targeted therapy (Hirsch et al., 2003; Krause and Van Etten, 2005; Nicholson et al., 2001; Passaro et al., 2021a). Exon 19 deletion (19del) and exon 21 L858R point mutations predominate the mutants sensitive to EGFR tyrosine kinase inhibitors (TKIs) (Passaro et al., 2021b) and have been widely investigated in many prospective clinical trials. Rare mutations together account for approximately 10% of *EGFR* alternations in NSCLC, of which exon 20 insertions, G719X in exon 18, S768I in exon 20, L861Q in exon 21, and dual mutations are the most predominant types. Although rare mutations occur at relatively low frequencies in lung cancer, the overall high incidence of lung cancer leads to a significant number of patients affected by these uncommon mutations, contributing to a substantial medical burden due to the need for specialized treatment strategies for these specific mutations (Harrison et al., 2020).

Compared to common mutations of *EGFR*, limited survival data of EGFR-TKIs are available for NSCLC patients with uncommon mutation sites (Gristina et al., 2020). Additionally, heterogeneous responses are usually observed in retrospective studies, causing the lack of treatment reference in first-line clinical decisions (Asahina et al., 2006; De Pas et al., 2011; Watanabe et al., 2014; Wu et al., 2011). Recent advances have revealed that afatinib, the second-generation TKIs approved for first-line treatment of NSCLC, shows clinical activity against many rare *EGFR* mutations, especially for highly detectable G719X, L861G, and S768I mutations (Yang et al., 2015; Yang et al., 2020). Furthermore, preclinical data suggests that these major uncommon mutations are more sensitive to second- and third-generation EGFR-TKIs rather than first-generations (Kobayashi and Mitsudomi, 2016). While other subtypes of rare mutations are far less studied. L858M, the amino acid substitution at exon 21, can be identified in 1%–2% EGFR-positive NSCLC patients (Tam et al., 2006). It has been reported that approximately 5% of NSCLC patients have L861R mutations (Abdelmaksoud-Dammak et al., 2022). Based on 15 cases with dual *EGFR* mutations, researchers have found the existence of L858M/L861Q in two cases, whereas no co-mutations of L858R and L861Q are detectable, suggesting that L858M rather than L858R serves as the partner of mutations occurring on 861 residues (Tam et al., 2006). However, there have been few studies on the dual L858M/L861R mutations and their durable response against TKIs, limiting the establishment of standard care in practice.

Here, we present the first report of administrating afatinib plus anlotinib in an advanced NSCLC patient with rare *EGFR* L858M/L861R mutations. Follow-up data have demonstrated remarkable efficacy and safety of this combination therapy, providing valuable insights into the clinical management of NSCLC patients with uncommon *EGFR* mutations.

## 2 Case presentation

**FIGURE 1:**
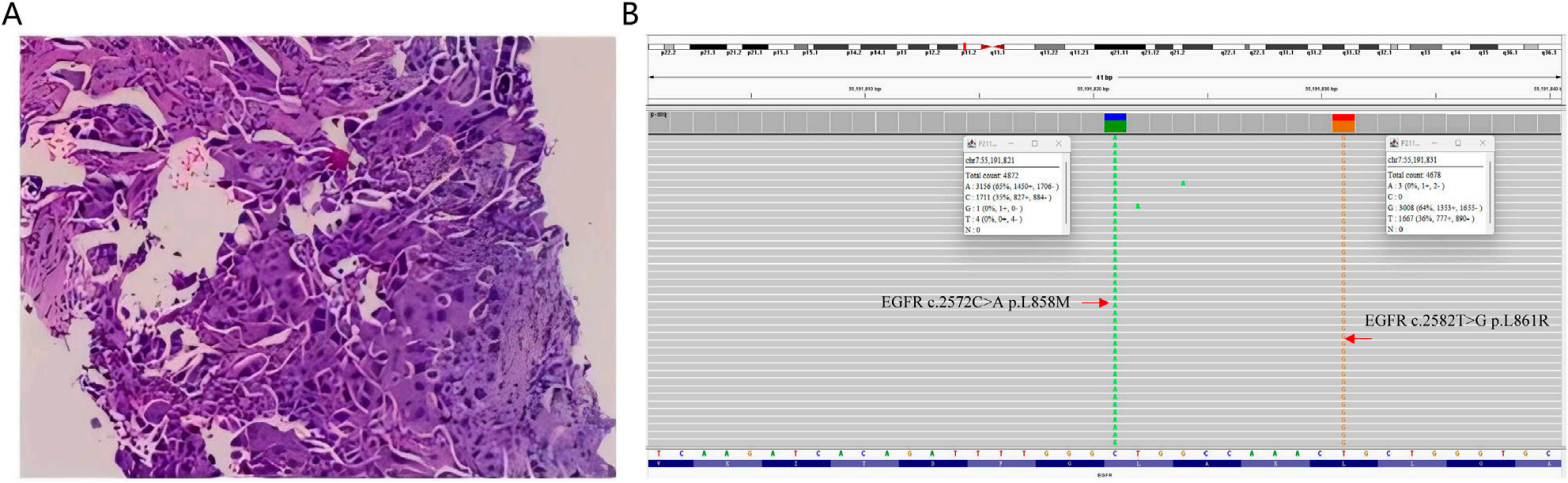
**(A)** Histopathological examination of the punctured lymph node in right supraclavicular derived from the patient. **(B)** Integrative Genomics Viewer visualizing the presence of compound *EGFR* L858M (c.2572C>A) and L861R (c.2582T>G) mutations in the patient’s tumor sample.

**FIGURE 2:**
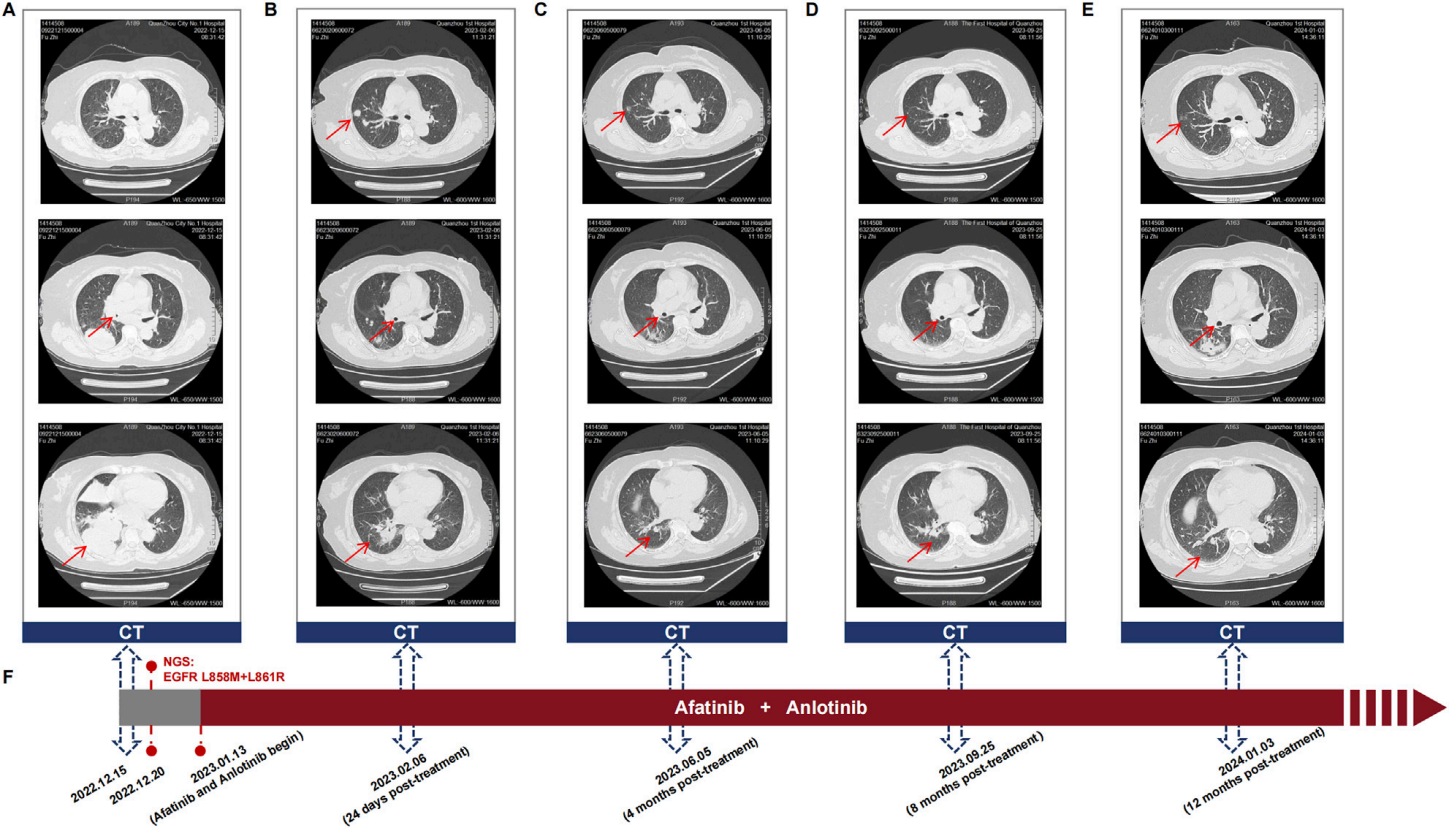
**(A–E)** Sequential chest CT scans showing the response of the primary lung tumor and metastatic lesions to the combination therapy of afatinib and anlotinib in the patient with *EGFR* L858M/L861R mutations. These scans were taken at **(A)** diagnosis, **(B)** 24 days post-treatment, **(C)** 4 months post-treatment, **(D)** 8 months post-treatment, and **(E)** 12 months post-treatment. Upper, middle, and lower CT images indicate the changes in tumor size, bronchial obstruction, and atelectasis, respectively. **(F)** Timeline of the patient’s treatment course, highlighting key events of diagnosis, mutation detection, initiating afatinib and anlotinib treatment, and follow-up visits.

On December 15, 2022, a 65-year-old female was admitted to the hospital due to recurrent cough and sputum for more than 2 months, without smoking, drinking, and other medical history. Physical examination showed an enlarged lymph node at the approximate size of 3.7*2.1 cm in the right supraclavicular, with slightly hard texture, poor mobility, and clear boundary. Chest CT revealed a right inferior lobe mass with peripheral obstructive pneumonia, atelectasis of right middle lobe, and multiple lymph node metastases in the right hilus pulmonis, mediastinum, and right supraclavicular (Figure 2A). Serum levels of carcinoembryonic antigen (CEA), carbohydrate antigen 15-3 (CA15-3), CA125, and squamous cell carcinoma antigen (SCC-Ag) were 13.57 ng/mL, 96.6 U/mL, 44.2 U/mL, and 62.39 ng/mL, respectively, indicating elevated tumor biomarkers compared to healthy individuals. The pathological examination of punctured lymph node in right supraclavicular confirmed NSCLC (Figure 1A). Additionally, immunohistochemistry reported positive expressions of TTF1, BRG1, CK7, CKP, and p63, indicative of a poorly differentiated metastatic adenocarcinoma originating from the lung. The whole-body bone scan showed no obvious abnormalities. Taken together, the patient was diagnosed with right inferior lobe adenocarcinoma, classified as cT3N3Mx IIIC stage.

Molecular characterization of a tumor tissue sample was conducted utilizing a 71-gene next-generation sequencing (NGS) panel (Repugene Technology, Hangzhou, People’s Republic of China), identifying rare *EGFR* L858M (c.2572C > A) and L861R (c.2582T > G) mutations that have been reported to account for 1%–2% of EGFR-positive cases (Figure 1B) (De Pas et al., 2011; Tam et al., 2006). Additionally, *EGFR* amplification, *RB1* mutation, and *TP53* mutation were detected, and PD-L1 was highly expressed. Based on a literature review and clinical experience in practice, combined treatment of afatinib (30 mg once daily) and anlotinib (10 mg once daily) was employed from January 13, 2023, with the patient’s consent. Twenty-four days after treatment initiation, notably, color ultrasound showed that the lymph node located in right neck was reduced compared to its pre-treatment dimensions, and the maximal size of post-treatment lymph node was 1.8*1.6 cm approximately. Moreover, chest CT scans demonstrated a continuous reduction of the primary tumor lesion on the right side, leading to an efficacy evaluation of a partial response, demonstrating positive drug efficacy. A novel lump was discovered in the left lobe (Figure 2B). Given its location, it raises the consideration that it may have developed during the interim between the initial imaging at admission and the start of the treatment, suggesting emergence of the lump is due to the gap in imaging sessions rather than a rapid progression after initiating treatment.

**FIGURE 3:**
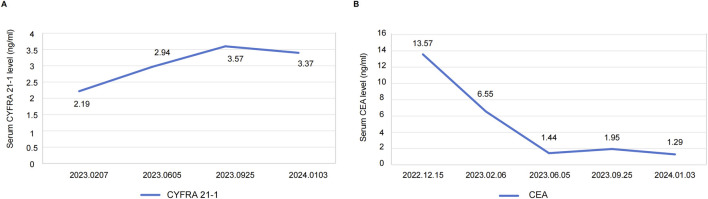
**(A)** Serum levels of CYFRA21-1 from 25 days post-treatment (2023.0207) to the latest visit. **(B)** Serum levels of CEA at the time of diagnosis (2022.12.15) and each follow-up visit. Definition of abbreviations: CYFRA 21-1 = cytokeratin-19 fragment; CEA = carcinoembryonic antigen.

After 4 months of treatment, this positive drug response was further evidenced by the decrease in lymph node size and reduced dimensions of primary lesions (Figure 2C). Although the patient experienced mild hand-foot syndrome and intermittent diarrhea after 3–4 months of combination therapy, these symptoms were effectively alleviated with the administration of appropriate medications. On September 25, 2023 (8 months after treatment), color ultrasound and CT uncovered stable sizes of lymph nodes and tumor mass, respectively (Figure 2D). Based on the recent follow-up information in early January (nearly 12 months post-treatment), no enlarged lymph node was observed, and primary tumor lesions exhibited further shrinkage (Figures 2E, F). Apart from minor hand-foot syndrome and intermittent diarrhea, the patient experienced no other adverse reactions or notable damage to the liver and kidneys. Cytokeratin-19 fragment (CYFRA 21-1), another serum tumor biomarker, is often abnormally elevated in NSCLC patients and is commonly utilized in clinical management (Takada et al., 1995). Promisingly, the serum levels of CYFRA 21-1 approximately remained within normal limits from 25 days post-treatment to the latest visit (Figure 3A). CEA declined compared to the penultimate follow-up point, almost keeping the levels within normal limits (Figure 3B).

Taken together, our therapeutic strategy combining afatinib and anlotinib has demonstrated satisfactory efficacy in a patient carrying rare L858M/L861R mutations. At the time of writing this report, this patient continues to receive the treatment and shows a positive response, maintaining a good quality of life. Follow-up examinations will be regularly conducted to monitor the patient’s progress. The evaluation of efficacy and adverse events was performed based on RECIST (Eisenhauer et al., 2009) and CTCAE standards, respectively.

## 3 Discussion

In this study, we identified the co-existence of rare *EGFR* L858M and L861R mutations in a treatment-naive female patient using an NGS test. Through a literature review, we found that only one case had previously reported the presence of dual L858M and L861R (Hong et al., 2017), though compound mutations comprising L858M or L861R with other mutant sites have been detected in NSCLC individuals (Bejjanki et al., 2017; Berge et al., 2013; Saxon et al., 2017; Zhang et al., 2020). Regrettably, this patient suffered tumor progression after 3 months of initiating gefitinib treatment, with a short overall survival (OS) of 7 months (Hong et al., 2017). The lack of reliable reference for determining first-line therapy in patients with dual L858M and L861R mutations posed a great challenge for us in making treatment decisions, but it also spurred us to search for an alternative therapeutic approach.

Given its unfavorable efficacy in targeting L858M/L861R, gefitinib was excluded as a first-line treatment option (Hong et al., 2017). A study reported that a non-smoker NSCLC patient receiving erlotinib showed disease progression by imaging and developed G719D plus L861R mutations after 9 months of treatment. This patient continued with erlotinib after relapse but opted out after another 2 months, making it challenging to comprehensively evaluate the efficacy and safety of erlotinib in L861R-mutated patients (Berge et al., 2013). In another study, resistance against erlotinib and rociletinib occurred in a young woman with an initial molecular diagnosis of primary L858M and L861Q mutations (Bejjanki et al., 2017). Erlotinib was also reported to induce severe drug toxicity within 4 months of treatment in a patient with *EGFR* L858M/L861Q mutations, including skin injury and shortness of breath (Saxon et al., 2017). Therefore, erlotinib may not be an optimal choice for patients carrying either L858M or L861R mutations. Osimertinib has shown superior efficacy in overcoming common resistant mutations (such as T790M) following treatment failure with first- and second-generation TKIs, prompting us to keep osimertinib as a sequential therapeutic strategy rather than a first-line clinical option (Mok et al., 2017; Passaro et al., 2017).

Notably, it seemed that patients with L858M or L861R mutations benefited from second-line oral afatinib treatment. A female patient with dual *EGFR* L858M and L861Q mutations switched to afatinib after the failure of initial erlotinib treatment, and obtained a significant clinical response for more than 10 months. Meanwhile, *in vitro* analysis has shown that L858M/L861Q mutated cell lines treated by afatinib displayed significantly impaired *EGFR* L858M/L861Q phosphorylation compared with the ones treated with gefitinib and osimertinib (Saxon et al., 2017). The clinical activity of afatinib in targeting L858M/L861Q mutation is further supported by evidence from a patient who benefited from a 25-week treatment of combining afatinib and cetuximab (Bejjanki et al., 2017). Moreover, durable response of afatinib was observed in another TKI-pretreated case with L858M (Yang et al., 2020). Despite limited cases reporting the application of afatinib in treatment-naive cohorts with L858M or L861R mutations, a brief report highlighted a remarkable 16-month of PFS in an NSCLC patient with S720F/L861R mutations after initiating afatinib monotherapy (Zhang et al., 2020). Considering its superior efficacy across a broad range of rare genetic sites and its relatively durable activity in L858M or L861R mutated tumors either in TKI-naive or TKI-pretreated patients, afatinib was selected as the primary clinical option in this study.

In addition to *EGFR* L858M/L861R mutations, the patient in this study was also diagnosed with *EGFR* amplification, *RB1* mutation, and *TP53* mutation. Given the relatively high mutation burden and large tumor lesions, combination therapy was prioritized. The critical role of angiogenesis in tumor progression and invasion highlights the potential of mediators involved in angiogenesis as promising candidate targets, one of which is the vascular endothelial growth factor receptor (VEGFR) (Alvarez-Aznar et al., 2017; Byrne et al., 2005; Folkman, 1971). The fact that VEGFR and EGFR signaling pathways share many downstream targets has led to the hypothesis that silencing VEGFR may effectively suppress bypass activation during EGFR-TKI treatment. Most recently, preclinical studies and clinical trials have demonstrated the synergistic anti-tumor effects of antiangiogenic agents and targeted therapy, significantly prolonging PFS of advanced NSCLC patients (Horn and Sandler, 2009; Pennell and Lynch, 2009; Saito et al., 2019; Tabernero, 2007). As a novel multitarget TKI for tumor angiogenesis, anlotinib exhibits the potential in reversing resistant tumors and serving as a posterior-line therapeutic strategy (Han et al., 2018). The combination of anlotinib with gefitinib or osimertinib significantly restored therapeutic sensitivity and inhibited the proliferation of resistant lung cancer cells *in vitro* and *in vivo* experiments (Lei et al., 2023; Li et al., 2021). Of note, an increasing number of randomized clinical trials are being conducted and have revealed the promising effects of anlotinib together with EFGR-TKIs, including icotinib (Zhang et al., 2023), erlotinib (Chu et al., 2022), gefitinib (Zhang et al., 2021), osimertinib (Han et al., 2022), and almoertinib (Chen et al., 2023), for untreated EGFR-positive NSCLC patients. These findings suggest that the combinations of anlotinib with EGFR-TKIs may exhibit superior efficacy and are encouraged as first-line options.

After an extensive literature review, we ultimately applied a combination therapy of afatinib and anlotinib for treating an NSCLC patient harboring compound *EGFR* L858M/L861R mutations, with the patient’s consent. To date, this approach has shown promising efficacy and survival outcomes, with no significant severe adverse effects or apparent damage to the liver and kidney functions but only mild hand-foot syndrome and diarrhea. This patient has been undergoing treatment for exceeding 12 months with PFS not yet reached, which is superior to the 3 months of PFS and 7 months of OS in a gefitinib-treated patient with dual L858M/L861R mutations. The outstanding efficacy and good safety profile of our case indicate that the combination therapy of afatinib and anlotinib can be considered as a viable treatment option for patients with rare *EGFR* mutations, specifically L858M/L861R compound mutations in NSCLC.

## 4 Conclusion

This study presents a patient of NSCLC diagnosed with dual *EGFR* L858M/L861R mutations, a compound subtype that has been previously reported once before. Through a thorough review of similar cases and efficacy comparison, we applied a first-line treatment of afatinib combined with anlotinib. Superior efficacy demonstrated the clinical activity of afatinib and anlotinib in overcoming L858M/L861R mutations, indicating the candidate therapeutic strategy in overcoming compound rare mutations in NSCLC. This report not only highlights the potential of this therapeutic approach but also reinforces the significance of tailoring treatment strategies in improving patient care in oncology, especially for those with atypical genetic profiles.

## Data Availability

The original contributions presented in the study are included in the article/supplementary material, further inquiries can be directed to the corresponding author.
